# Factorial design-assisted supercritical carbon-dioxide extraction of cytotoxic active principles from *Carica papaya* leaf juice

**DOI:** 10.1038/s41598-018-37171-9

**Published:** 2019-02-08

**Authors:** Kooi-Yeong Khaw, Marie-Odile Parat, Paul Nicholas Shaw, Thao T. T. Nguyen, Saurabh Pandey, Kristofer J. Thurecht, James Robert Falconer

**Affiliations:** 1 School of Pharmacy, The University of Queensland, Brisbane, QLD 4072 Australia; 2 The Centre for Advanced Imaging (CAI), The University of Queensland, Brisbane, QLD 4072 Australia

## Abstract

The aims of this study are to investigate the selective cytotoxic activity of supercritical carbon dioxide (scCO_2_)-extracted freeze-dried leaf juice (FDLJ) of *Carica papaya* on squamous cell carcinoma (SCC25) cells, and to delineate the best small scale extraction parameters allowing maximal extract activity. Using scCO_2_ as a solvent, six operating parameters were studied and the supercritical fluid extraction (SFE) process investigated using a factorial design 2^6-2^. The processing values promoting cytotoxic activity towards SCC-25 are: high pressure (250 bar), low temperature (35 °C), extended processing time (180 minutes), as well as a large amount of starting material (5 g). The factorial experimental design successfully identified the key parameters controlling the SFE of molecules cytotoxic to SCC cells from *C. papaya* juice. This study also validated the extraction method and showed that the SFE yield was reproducible. The chromatographic and mass spectrometric profiles of the scCO_2_ extract acquired with high-resolution quadrupole time-of-flight mass spectrometry (LC-QToF-MS) were used to tentatively identify the bioactive compounds using comparative analysis. The principal compounds were likely to be mainly vitamins and phytosterols, some of which are documented to be cytotoxic to cancer cells.

## Introduction

In the context of increasing demand for natural products, more effective and selective extraction methodologies are required for the rapid recovery of pharmacologically active compounds from raw plant materials^1^. The supercritical fluid extraction (SFE) method has emerged as a highly selective alternative to standard solvent-based techniques^2,3^. The principle of supercritical fluid (SCF) as an extraction method is based on the properties of a substance, where the pressure and temperature are above its critical point, forming a homogenous phase with liquid and gas-like properties^4^. The intricate fluid dynamics of complex mixtures and SFE are still poorly understood, thus to date, there is no standard method that accommodates a wide range of starting raw material. Furthermore, carbon dioxide, the most common SCF, when used purely as a non-polar solvent, is incapable of dissolving polar compounds; even large hydrocarbon compounds (>1000 Da) with strong ionic functional groups, are polar and, as such, cannot be extracted. This partly explains the ‘selective nature’ of supercritical carbon dioxide (scCO_2_) when used in SFEs. There are ‘polar soluble’ SCFs such as dimethyl ether (DME) and water, however DME is toxic to humans if inhaled, requiring worker and environmental protection measures, while water requires large amounts of energy to reach its critical point of at least 374 °C (705 °F) and 218 bar (~3200 psi). The actual optimal extraction temperature and pressure could well be much higher than these values and hence, all thermo-liable compounds that degrade at around 374 °C or less would be destroyed. Where the use of co-solvents (e.g. ethanol) or surfactants is not desirable, scCO_2_ is the preferred solvent. To understand better the complicated solvent-solvent, solvent-solute, and solute-solute interactions of scCO_2_ under high pressures, a typical solution is to conduct a full range of experiments to generate sufficient information to identify the optimal conditions of SFE^5^.

A number of conditions referred to as factors herein, have been proposed to influence SFE. For example, and most obviously, pressure and temperature, but also solvent flow rate, sonication, raw material preparation (crushing/drying/attrition), stirring rate, extraction time, starting amount of raw materials, and if desirable, the use of co-solvents and their characteristics and amounts. Due to the number of factors that can be fine-tuned to optimize the extraction of active principles, a formal full factorial experimental approach is impractical, especially at the screening stage.

Mathematical models can be used to dramatically reduce the number of experiments required to screen a large number of factors (≥4) simultaneously. Fractional Factorial Designs (FFDs) can also provide information on potential high-order interactions, something that is impossible using a full factorial method – measuring one-factor-at-a-time. Due to this ability to detect interactions between multiple factors, FFDs are to a large extent less susceptible to outliers than full factorial designs. Should FFD-based screening raise questions about certain factors, further investigations can be made adding to existing data and working towards full factorial information on those specific factors, that is sequential experimentation. Another advantage of fractional screening is that there are fewer experiments conducted, thereby significantly reducing the time required to acquire information and lowering running costs for materials^6^. At a higher level, the use of FFDs is justified based on the ‘sparsity of effects’ principle, which states that: (i) there may be many factors affecting a system, but usually only a few are important and, (ii) interactions between factors are low. There are clear limitations with fractional factorial designs (FFDs), however, where there are multiple factors known to be important and the project work is at the proof-of-concept level, then FFD use is warranted.

In this study, the FDLJ of tropical plant *C. papaya* found in Australia is subjected to a SFE method employing carbon dioxide as a solvent according to a FFD model of experimentation. This plant is well-known within tropical and sub-tropical regions. The fruit is consumed worldwide and used in a range of products such as cream, oil and processed food^7^. The papaya leaf has been used in an attempt to treat dengue fever, malaria, as well as cancer^8–11^. Other parts of *C. papaya* including its bark, roots, latex, flower, and seeds have been used as a traditional treatment of many different illnesses^12^. Recent research has demonstrated that the leaf extracts of *C. papaya* possess interesting anti-cancer properties against breast, oral squamous, and pancreatic cell lines^11,13,14^. In particular, selective cytotoxicity on cancer cells sparing non-cancer cells has been documented for FDLJ^15^. The aim of the present study was to identify and partially-optimise factors affecting SFE yield and extract actives cytotoxic towards a SCC25 cell line. In addition, the selectivity of the SFE extracts towards the cancerous SCC25 cell line in comparison to a non-cancerous human keratinocyte (HaCaT) cell line was investigated. We further aimed to carry out preliminary identification bioactive compounds by LC-QToF-MS.

## Materials and Methods

### Chemicals and reagents

Dulbecco’s Modified Eagle’s Medium (DMEM), DMEM-F12, penicillin/streptomycin, trypsin, and foetal bovine serum (FBS) were purchased from Invitrogen (Life Technologies, Mulgrave, VIC, Australia). 3-(4,5-dimethylthiazol-2-yl)-2,5-diphenyltetrazolium bromide (MTT), dimethyl sulfoxide (DMSO), analytical grade ethanol, and HPLC grade methanol were obtained from Merck (Darmstadt, Germany). Purified water was generated using a MilliQ system (Millipore, Bilerica, MA, USA). Carbon dioxide (>99.9) was obtained from BOC (Sydney, Australia).

### Preparation of papaya FDLJ

Organically grown *C. papaya* leaves were collected from Tropical Fruit World, a privately owned plantation and Research Park in northern New South Wales (NSW), Australia. Papaya leaves were washed under running tap water to remove as many contaminants as possible, and rinsed with MilliQ water to obtain clean leaves. Batches of approximately 934 g of leaves were juiced using a Green-power juice extractor (Korea) without the addition of water. The leaf juice was lyophilised using a Christ Alpha 2-4LD freeze-dryer (Martin Christ Gefriertrocknungsanlagen GmbH, Germany) to obtain a greenish coloured powder that was then stored at −80 °C. The yield of the leaf juice preparation was 9.35 ± 0.88% w/w (n = 10).

### SFE equipment and setup

**Figure 1:**
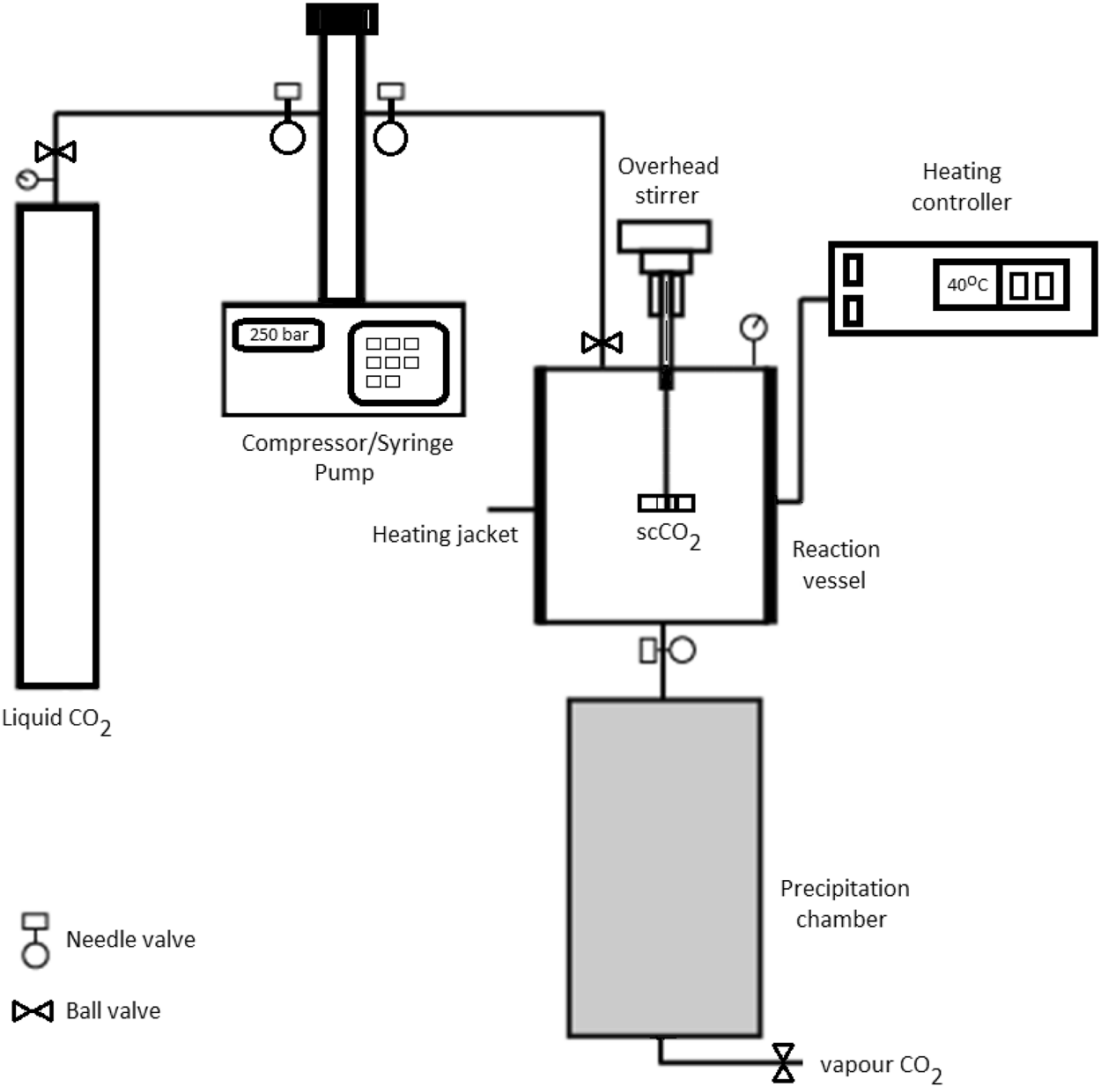
Schematic of the supercritical fluid (SCF) extractor.

Supercritical fluid extractions were carried out using a laboratory scale extraction system. The SFE system consisted of a liquid carbon dioxide reservoir, high pressure syringe pump (Teledyne Isco 260D), 60 mL stainless steel 316 extraction vessel, precipitation chamber, backpressure regulator and an overhead stirrer. Figure 1 shows a schematic of the SFE system. Glass wool was placed in front of the outlet tube on the inside of the extraction vessel and held there using a stainless steel mesh to prevent entrainment of the sample.

A 150 Watt heating jacket (WatLow, USA) was used around the extraction vessel to maintain the desired temperature. A 250 W sonication bath (Grant, United Kingdom) was used in this study. The separation step involved opening the valve to the precipitation chamber via a capillary nozzle (1/16″ diameter) and the CO_2_ (gas) was discharged to the atmosphere leaving solvent-free extract. The extract was collected in a vial by washing the precipitation chamber with ethanol (10 mL). The solution was dried using nitrogen gas to evaporate the ethanol. The extracts were weighed to determine the extraction yield;

1 $$ \% \,{\rm{yield}}={\rm{Y}}/{\rm{Yo}}\times 100$$

where Y is the weight of dried extract and Yo is the weight of the sample.

### SFE processing and experimental domain

Using the SFE system described above, liquid CO_2_ was pumped and compressed in the extraction vessel and heated according to the conditions listed in Table 1. Dried extracts were stored at −20 °C until required for further testing. The extraction process is principally affected by the density and diffusivity of the SCF, therefore, pressure and temperature, respectively are important factors. Table 1 shows the selected factors for the experimental domain. Six factors were investigated including pressure (A), temperature (B), processing time (C), amount of starting material (D), sonication time (E) and stirring rate (F) at low and high values. The range of pressures employed (85–250 bar) was used in the supercritical region for carbon dioxide and there was no sub-critical experiments performed. The temperatures used were in the range of 35–50 °C, to reach SCF temperature conditions and preserve thermally labile compounds from degradation over 50 °C. Processing intervals of 30 minutes (low) and 180 minutes (high), starting material amounts of 1 g and 5 g, effects of sonication (absent-low and 30 min-high) as well as the stirring rate (absent-low and 450 rpm-high) were evaluated. All experiments were performed according to the factorial design created in Minitab 17 (see Sections 2.5 to 2.8). The measured responses were defined as the percentage of cytotoxicity to SCC25 cancer cell line (see Sections 2.9 to 2.11) in comparison to unexposed cells. The experimental domain is presented in Table 1.

**Table 1:** Selected parameters for the experimental domain.

Factor	Low level (−)	Centre point (0)	High level (+)
A Pressure (bar)	85	150	250
B Temperature (°C)	35	43	50
C Processing time (min)	30	120	180
D Material (g)	1	3	5
E Sonication time (min)	absent	15	30
F Stirring rate (rpm)	absent	200	450

### Experimental matrix

The factorial design of 2^6-2^ was employed to give an 18 run experimental plan with two centre points positioned at a medium level between the set low and high levels. Having centre points is used as a reference and helps to determine the factor-response linearity and experimental error. The experimental matrix is given in Table 2 together with the factors for A, B, C, D, E and F as listed above in Section 2.4.

**Table 2:** Experimental matrix for the 2^6-2^ design for the extraction.

SFE processing parameters						
Runs	Factors					
	A	B	C	D	E	F
1	−1	−1	−1	1	−1	1
2	−1	1	1	−1	−1	−1
3	−1	−1	−1	−1	−1	−1
4	−1	1	1	1	−1	1
5	−1	−1	1	−1	1	1
6	0	0	0	0	0	0
7	1	−1	−1	1	1	1
8	1	−1	−1	−1	1	−1
9	1	−1	1	−1	−1	1
10	−1	−1	1	1	1	−1
11	1	1	−1	−1	−1	−1
12	1	−1	1	1	−1	1
13	−1	1	−1	1	1	1
14	1	1	1	−1	1	1
15	0	0	0	0	0	0
16	1	1	−1	1	−1	−1
17	−1	1	−1	−1	1	1
18	1	1	1	1	1	1

A = pressure, B = temperature, C = processing time, D = material.

E = sonication time and F = stirring rate. Row 6 and 15 are the central points.

### Resolution

The factors A to D of the experimental matrix form a full factorial design. Factors E and F are estimated using statistical modelling known as generators. These were formed by multiplying the previous four-factor columns. That is E = A.B.C, and F = B.C.D and the generating relations can be expressed as;

$$[I={\rm{ABC}}{\bf{E}};{I}={\rm{BCD}}{\bf{F}};{I}={\rm{AD}}{\bf{E}}{\bf{F}}]$$

The shortest word in the generating relations is four, producing a resolution IV experimental design. This level of resolution provides confounding information of factors.

### Regression modelling

Regression analysis provides statistical estimation between responses and independent factors. A multiple regression model can be represented by the equation;

2 $$\begin{array}{ccc}{Y}_{j} & = & {\beta }_{0}+{\beta }_{1}{\rm{A}}+{\beta }_{2}{\rm{B}}+{\beta }_{3}{\rm{C}}\,+\,{\beta }_{4}{\rm{D}}\,+\,{\beta }_{5}{\rm{E}}\,+\,{\beta }_{6}{\rm{F}}\,+\,{\beta }_{7}{\rm{AB}}+{\beta }_{8}{\rm{AC}}+{\beta }_{9}{\rm{AD}}\\  &  & +\,{\beta }_{10}{\rm{AE}}+{\beta }_{11}{\rm{AF}}+{\beta }_{12}{\rm{BD}}+{\beta }_{13}{\rm{BF}}+{\beta }_{14}\mathrm{ABD}+{\beta }_{15}\mathrm{ABF}+{\rm{\varepsilon }}\end{array}$$

where,

- *Y* = Estimated response of experiment j
- *β*_0_ = Coefficient constant of the average experimental response*
- *β*_1_ to *β*_6_ = Estimated main effects of variables
- *β*_7_ to *β*_15_ = Estimated interaction effects of variables
- A to F = Effect variables
- ε = Experimental error term

*also known as the grand mean, ($\mathop{Y}\limits^{\_}={\rm{T}}/{\rm{N}}$), where T is the grand sum and N is the sample size.

### Statistical analysis of the experimental design

The design of experiment (DOE) for the factorial runs and data analysis was performed with Minitab V16.0 (Minitab Inc., State College, PA, USA). Statistical significance was considered to be *P < 0.05.

### Sample preparation for cytotoxicity assay

For cytotoxic activity determination, FDLJ extracts (from SFE) were prepared in a series of concentrations (representing 1–100 mg/mL of original leaf) in serum-free culture medium for cytotoxic activity determination. The papaya extract-containing samples were filtered through a 0.22 μm sterile filter (JETBIOFIL) and stored at 4 °C before performing the test.

### Cell culture

SCC25 cells were cultured in Dulbecco’s Modified Eagle’s Medium (DMEM)/F12 medium supplemented with 10% v/v heat-inactivated foetal bovine serum (GIBCO), penicillin (100 units/mL) and streptomycin (100 μg/mL) (Invitrogen) and 0.4 μg/mL hydrocortisone. HaCaT cells were cultured in Dulbecco’s Modified Eagle’s Medium (DMEM) medium supplemented with 10% v/v foetal bovine serum (GIBCO) and penicillin (100 units/mL) and streptomycin (100 μg/mL) (Invitrogen). Both cell lines were grown in a humidified incubator with 5% CO_2_ at 37 °C. The cultures were allowed to grow until approximately 70–90% confluent and experiments were performed.

### MTT assay

Cells (6,000 SCC25 cells per well and 3,000 HaCaT cells per well) were seeded into a 96-well plate (GREINER BIO-ONE). After 24 hours, the medium was removed and 100 μL of serum-free medium containing different concentrations of extracts were added and incubated for 48 hours at 37 °C. The medium was replaced with a MTT solution (0.5 mg/mL) in serum-free culture medium. After incubation for 2 hours, the medium was removed from the wells and the formazan crystals trapped in cells were dissolved in 100 μL of DMSO by shaking for 20 min on an orbital shaker. The absorbance values were measured at 595 nm using a Lmark plate reader (BioRad, USA). The absorbance of blank wells containing no cells was subtracted, and the absorbance of wells where the cells were exposed to control medium taken to be 100% cell survival. Results were calculated as the percentage of viable cells compared to the control. For the factorial fractional design modelling, the results were expressed as the cytotoxicity, or 100 minus % of cell survival at the concentrations of extracts corresponding to 100 mg/mL of original leaves.

Statistical analysis and plotting of the data were performed with Prism 7 (GraphPad software Inc., San Diego, USA). All data were presented as mean ± SEM. Two-way ANOVA with Sidak post tests were used for comparisons of the activities on the two cell lines. One-way ANOVA with Dunnett’s multiple comparison tests was performed for selected experiments.

### LC-QToF-MS analysis

The system consisted of an Agilent 1290 UHPLC system (Agilent technologies, Santa Clara, CA, USA) together with an Agilent 6520 high-resolution accurate mass quadrupole time of flight (QToF) mass spectrometer equipped with a multisource for both electrospray Ionisation (ESI) and Atmospheric Pressure Chemical Ionization (APCI) modes. Chromatographic separation was performed using a 2.1 × 150 mm, 3.5 µm ECLIPSE PLUS C18 analytical column (Phenomenex, USA). Mobile Phase A was ultra-purified MilliQ water, while mobile phase B was HPLC grade methanol. The gradient elution condition was: 50% B for the first 5 min; 50–90% of B from 5–40 min; 90–100% of B from 40–60 min; 100–50% from 60–75 min. The flow rate was 0.2 mL/min and the sample injection volume was 5 µl. The run time was set at 75 minutes. MassHunter software (version B.02.01 SP3 –Agilent) was used to control the mass spectral acquisition. The operating conditions for mass spectrometer were m/z scan 100–1700, scan rate of 0.8 cycles/per second with the following conditions: nebuliser pressure 30 psi, drying gas flow 5.0 L/min, gas temperature 300 °C, fragmenting voltage 175 V and skimmer voltage 65.0 V.

### MS data analysis

Data analysis was performed using Agilent Mass hunter Qualitative software (version B.05.00 Agilent Technologies, Santa Clara, CA, USA, 2012) with molecular feature Extractor (MFE) algorithms with Mass Profiler Professional Software (Version 12.1, Agilent Technologies, Santa Clara, CA, USA, 2012) to align features from the chromatograms of scCO_2_ extract. The molecular feature generator algorithm was utilised to generate molecular formula from C, H, N, O, P and S. Compound identification was carried out with the METLIN personal metabolite database (>1 million metabolites including lipids, amino acids, carbohydrates, toxins, small peptides, and natural products)^16^.

## Results and Discussion

A summary of the cytotoxic effects of the FDLJ extracts of *C. papaya* produced by SFE is shown in Table 3. The results revealed that the activity of the extract produced from Run 12 was best (for the experimental domain investigated) when the processing pressure (A) was 250 bar, temperature (B) was 35 °C, processing time was 180 min, amount of material (FDLJ) was 5 g, and the stirring rate was 450 rpm. This SFE processing produced an extract with 67.3% toxicity (32.7% of cell survival) on SCC25 at a concentration of extract equivalent to 100 mg of original leaf material per mL of cell culture medium. The consistent response between the centre points (Run 6 and Run 15) indicated that the SFE process was reproducible.

**Table 3:** Experimental matrix for the 2^6-2^ design factors and responses for cytotoxicity.

Runs	Factors						Cytotoxicity* (R2)
	A	B	C	D	E	F	
1	−1	−1	−1	1	−1	1	10.12 ± 6.88
2	−1	1	1	−1	−1	−1	31.29 ± 4.70
3	−1	−1	−1	−1	−1	−1	2.17 ± 0.66
4	−1	1	1	1	−1	1	18.76 ± 7.7
5	−1	−1	1	−1	1	1	17.71 ± 7.76
6	0	0	0	0	0	0	24.78 ± 3.15
7	1	1	−1	1	1	1	24.62 ± 7.75
8	1	−1	−1	−1	1	−1	29.20 ± 6.92
9	1	−1	1	−1	−1	1	17.15 ± 1.62
10	−1	−1	1	1	1	−1	42.24 ± 3.68
11	1	1	−1	−1	−1	−1	18.69 ± 2.92
12	1	−1	1	1	−1	1	67.33 ± 5.30
13	−1	1	−1	1	1	1	19.55 ± 3.06
14	1	1	1	−1	1	1	28.81 ± 2.79
15	0	0	0	0	0	0	23.10 ± 2.82
16	1	1	−1	1	−1	−1	12.78 ± 3.98
17	−1	1	−1	−1	1	1	3.87 ± 1.76
18	1	1	1	1	1	1	11.78 ± 3.06

A = pressure, B = temperature, C = processing time, D = material, E = sonication time and F = stirring rate.

*% of cell death of SCC25 at a concentration equivalent to 100 mg original leaf material /mL cell culture medium (mean ± SEM) (n = 3).

Table 4 shows the summary of significant factors for the cytotoxicity studies. The coefficient of determination (R^2^) obtained from the calculated equation at 99.96%, shows a strong relationship among the factors chosen and cytotoxicity. The results were analysed for the standard error of the coefficients, t-values, P-values, and regression of coefficients.

**Table 4:** Summary of regression coefficients of the significant factors for cytotoxicity calculated with the stepwise method.

	Effect	Coefficient (SE)	t-value	P-value	R^2^
Cytotoxicity (R2) Constant		0.2977	74.74	0.009	99.96%
A	8.079	0.2977	13.57	0.047	
B	−8.127	0.2977	−13.65	0.047	
C	14.263	0.2977	23.95	0.027	
F	−13.832	0.2977	−23.23	0.027	
AB	−8.433	0.2977	−14.16	0.045	
BD	−12.232	0.2977	−20.54	0.031	

A = pressure, B = temperature, C = processing time, D = material, E atsonication time and F onstirring rate. Statistically significant factors (ANOVA, P-value ≤ 0.05).

### Model response - cytotoxicity

Table 5 shows the statistical parameters for the percentage of cell death of SCC25 cancer cells at 100 mg of original leaf material per mL of cell culture medium. All the parameters investigated, pressure, temperature, processing time and stirring rate had a significant influence on the cytotoxicity effect (P < 0.05). In addition, loading of material was included due to its borderline significance (P = 0.052). Processing time had the most profound effect on cytotoxicity, followed by stirring rate, temperature, pressure and loading of material.

**Table 5:** Estimated effects and coefficient for the processing cytotoxicity (R^2^ = 99.96%).

Term	Effect	Coefficient	SE-coefficient	T	P
Constant		22.255	0.2977	74.74	0.009
A	8.079	4.039	0.2977	13.57	0.047*
B	−8.127	−4.063	0.2977	−13.65	0.047*
C	14.263	7.131	0.2977	23.95	0.027*
D	7.286	3.643	0.2977	12.23	0.052*
E	−0.064	−0.032	0.2977	−0.11	0.932
F	−13.832	−6.916	0.2977	−23.23	0.027*
AB	−8.433	−4.217	0.2977	−14.16	0.045*
AC	−4.314	−2.157	0.2977	−7.24	0.087
AD	−1.621	−0.811	0.2977	−2.72	0.224
AE	−5.322	−2.661	0.2977	−8.94	0.071
AF	−2.634	−1.317	0.2977	−4.42	0.142
BD	−12.232	−6.116	0.2977	−20.54	0.031*
BF	4.003	2.001	0.2977	6.72	0.094
ABD	−4.902	−2.451	0.2977	−8.23	0.077
ABF	6.908	3.454	0.2977	11.60	0.055
Centre points		1.685	0.8932	1.89	0.310

A = pressure, B = temperature, C = processing time, D = material, E atsonication time and F onstirring rate. SE = standard error. Statistically significant factors (ANOVA, P-value ≤ 0.05).

A full regression model relating the cytotoxicity to the SFE processing conditions was generated from the factorial study and is shown in Equation 3;

3 $$\begin{array}{ccc}{{\rm{R}}}_{{\rm{cytotoxicity}}} & = & 22.255+4.039{\rm{A}}-4.063{\rm{B}}+7.131{\rm{C}}-3.643{\rm{D}}-0.032{\rm{E}}-6.916{\rm{F}}\\  &  & -4.217{\rm{AB}}-2.157{\rm{AC}}-0.811{\rm{AD}}-2.661{\rm{AE}}-1.317{\rm{AF}}-6.116{\rm{BD}}\\  &  & +2.001{\rm{BF}}-2.451{\rm{ABD}}-3.454{\rm{ABF}}+1.685\end{array}$$

The simplified model is presented in Equation 4;

4 $$\begin{array}{ccc}{{\rm{R}}}_{{\rm{cytotoxicity}}} & = & 22.255+4.039{\rm{A}}-4.063{\rm{B}}+7.131{\rm{C}}+3.643{\rm{D}}-6.916{\rm{F}}-4.217{\rm{AB}}\\  &  & -6.116{\rm{BD}}+1.685\end{array}$$

The ANOVA results for the refined model for the cytotoxicity is shown in Table 6. The larger the magnitude of F and the smaller the P-value, the greater the significance of the corresponding coefficient. The magnitude of the statistical significance factor is, in descending order: C > F > B > A > D. No curvature deviation was observed for the cytotoxicity effects from the model (P = 0.310).

**Table 6:** ANOVA table for refined model for the cytotoxicity.

Source	DF	SS	Adj SS	Adj MS	F	P
Main Effects	6	2316.53	2316.53	386.091	272.2	0.046
A	1	261.08	261.08	261.08	184.06	0.047*
B	1	264.17	264.17	264.17	186.25	0.047*
C	1	813.63	813.69	813.69	573.66	0.027*
D	1	212.31	212.31	212.31	149.69	0.052
E	1	0.02	0.02	0.016	0.01	0.932
F	1	765.27	765.27	765.26	539.52	0.027*
AB	1	284.47	284.47	284.47	200.56	0.045*
AC	1	74.43	74.43	74.43	52.48	0.087
AD	1	10.51	10.51	10.51	7.41	0.224
AE	1	113.31	113.31	113.31	79.88	0.071
AF	1	27.75	27.75	27.75	19.57	0.142
BD	1	598.47	598.47	598.47	421.93	0.031*
BF	1	64.09	64.09	64.09	45.19	0.094
ABD	1	96.10	96.10	96.10	67.75	0.077
ABF	1	190.87	190.87	190.87	134.56	0.055
Curvature		5.05	5.05	5.047		0.310

DF = degrees of freedom, F = F-test which has F-distribution.

under the null hypothesis. *Statistically significant parameters (ANOVA, P-value ≤ 0.05).

### Validation of the factorial model

Given pressure, temperature, processing time, and stirring were identified as statistically significant in terms of influencing the SFE process, they were selected for validation of the model. Furthermore, as the factor of raw material loading (5 g) had a P-value of 0.052, it was additionally included in the validation of the model, due to borderline statistical significance. The estimated cytotoxicity effects for the significant levels and minimised levels were, 58.5% and 6.6%, respectively. The actual cytotoxicity effect for the best model was 54.5 ± 6.5% which was considered sufficiently similar (93.2% of the best level) to render the model validated. The actual cytotoxicity of minimised levels was 9.2 ± 1.4%. This was quite different (28.3%) to the estimated cytotoxicity (6.6%) at the minimised levels for the model. Part of this difference is due to measuring smaller values, which means a small difference results in a large amount of variation between the model estimate vs. the actual effect. In addition, the general trend was that the minimised levels of the model did in fact bring about a markedly reduced cytotoxic effect, and the minimised levels are of lesser interest compared to that of the best levels.

### *In-vitro* cytotoxicity

**Figure 2:**
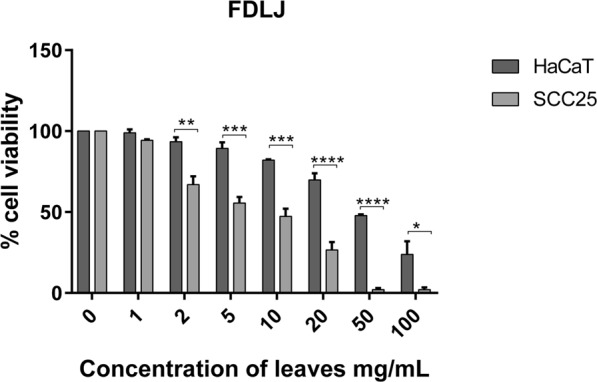
Effect of *C. papaya* FDLJ (control) on the survival of SCC25 and HaCaT cells. Results are shown as mean ± SEM (n = 3 independent experiments). *P < 0.05, **P < 0.01, ***P < 0.001, ****P < 0.0001, HaCaT vs SCC25 (two-way ANOVA with Sidak post-test).

**Figure 3:**
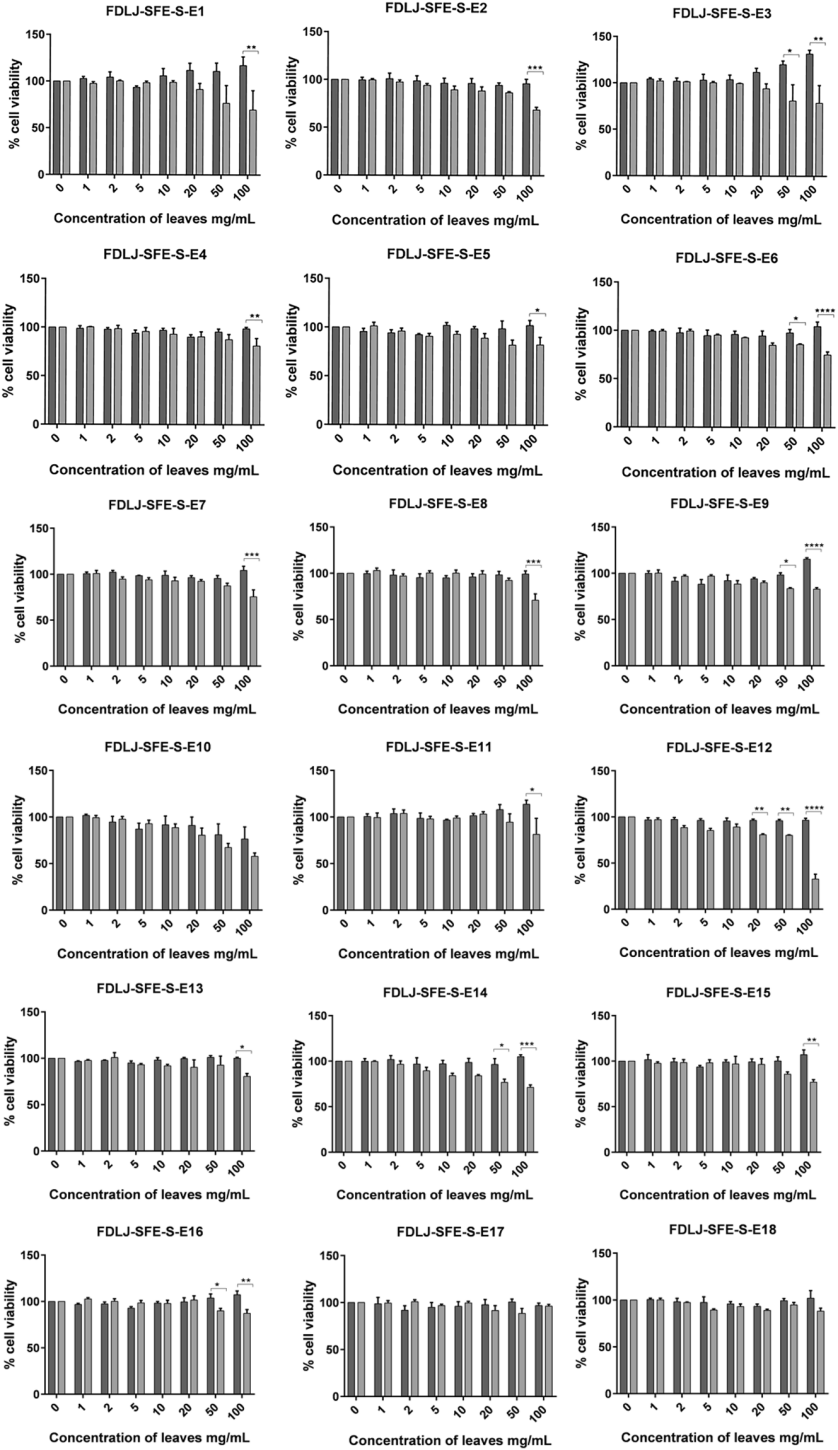
Effect of *C. papaya* FDLJ SFE extract on the survival of SCC25 and HaCaT cells. Results are shown as mean ± SEM (n = 3 independent experiments). *P < 0.05, **P < 0.01, ***P < 0.001, ****P < 0.0001, HaCaT vs SCC25 (two-way ANOVA with Sidak post-test).

Using FDLJ as a standard for comparison (Fig. 2), we evaluated the cytotoxicity and selectivity of the SFE extracts produced with the FFD. SFE extracts were screened for their cytotoxicity against SCC25 cells or HaCaT cells using the MTT method (Fig. 3).

The concentrations used ranged from 1 to 100 mg of original leaf material per mL of cell culture medium. At first glance, the FDLJ extract seemed more cytotoxic than the SFE extract, for example FDLJ-SFE S-E12. It is not surprising that some material has not been extracted in the process of extraction, it was actually anticipated, since we hypothesized that the SFE extract would keep some selective activity while being of a much simpler composition, thereby allowing analytical work and compound identification. While the FDLJ extract seemed more active than the SFE extract, the extent of its activity on HaCaT cells is not desirable in terms of clinical potential, and increases together with activity on SCC25. It is important to note that we have expressed our data by referring to the original leaf amount, which allows appropriate comparison, but it should be kept in mind that the extraction yields differed widely between FDLJ and SFE extraction. From the supplementary dataset 1: Table S1 shows the yield of SFE from FDLJ, Table S2 shows the statistical analysis, where none of the individual SCF processing parameters were calculated as significant, and Figure S3, shows the main effects plots for SFE processing yields from the parameters and levels investigated. Figure 3 shows the effect of FDLJ SFE extracts on cell viability of SCC25 cancer cells and HaCaT cells. Two-way ANOVA analysis of FDLJ-SFE-S-E12 showed that overall there were significant survival differences between the two cell lines (P < 0.0001). The Sidak multiple comparison test confirmed that FDLJ-SFE-S-E12 selectively affected the SCC25 cancer cells at concentrations in the range 20–100 mg/mL (Fig. 3). While FDLJ-SFE-S-E12 was less potent than the pre-SFE FDLJ (~33% survival vs ~2% survival, respectively, at a concentration equivalent to 100 mg leaves per mL medium), the data also showed that FDLJ-SFE-S-E12 was more selectively cytotoxic towards SCC-25 than control (FDLJ) at the concentration of 100 mg/mL of original leaf (P < 0.0001 *vs* P < 0.05). This may indicate that the SFE process permitted the extraction of molecules capable of selectively affecting the viability of the SCC25 cells. Other SFE extracts exhibited a weaker cytotoxicity effect against SCC25 cells with 57.7 to 97.8% cell survival. Interestingly, most SFE extracts either showed only very slight cytotoxicity to HaCaT cells, or no toxicity, or promoted their proliferation; this latter observation was most dramatic for FDLJ-SFE-S-E3 which statistically increased cell numbers (P = 0.0004, one-way ANOVA with Dunnett’s multiple comparisons test).

### Tentative identification of bioactive compounds by LC-QToF-MS of scCO2 extract of FDLJ

**Figure 4:**
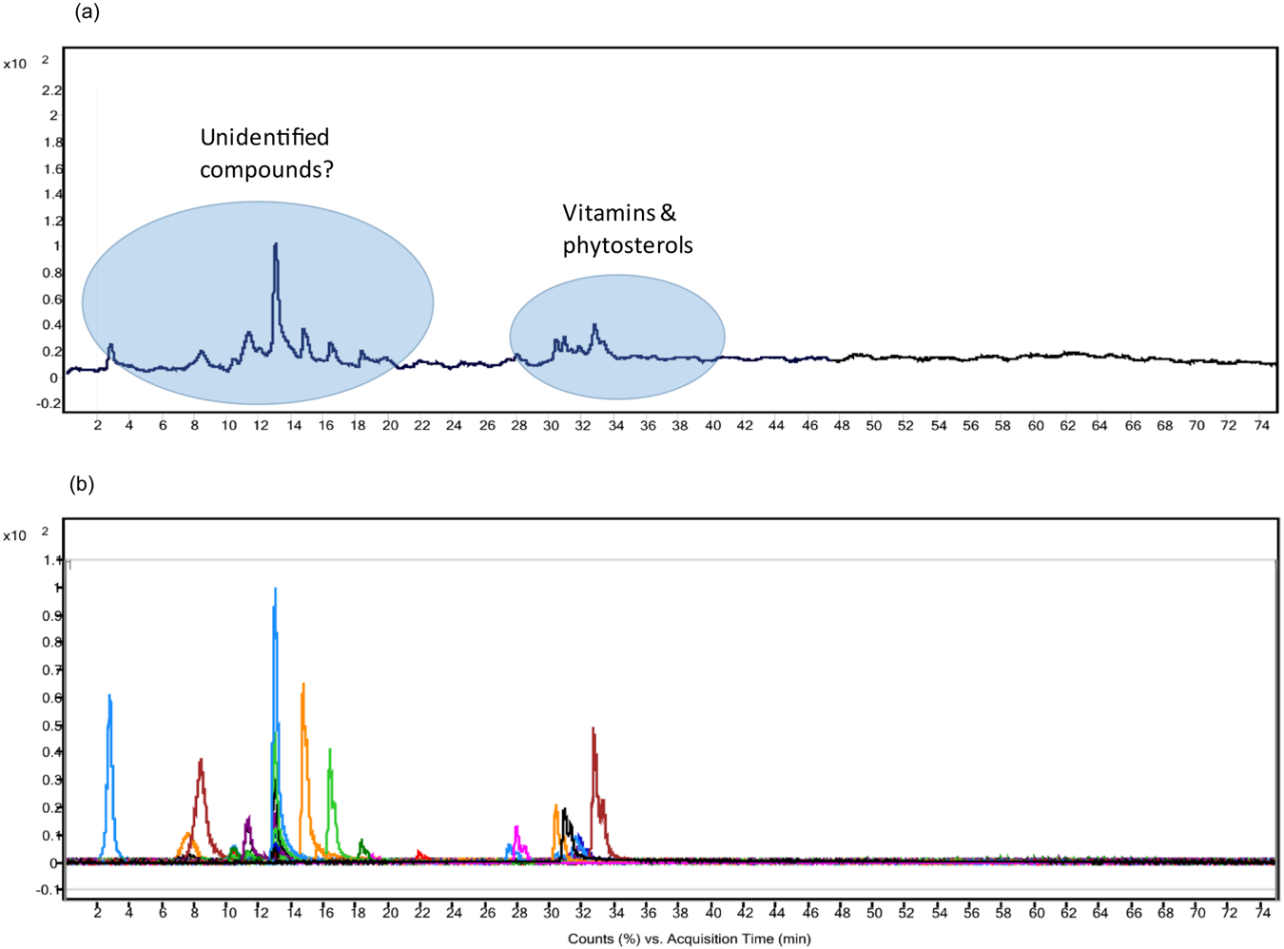
scCO_2_ extract of FDLJ profile acquired by LC-QToF-MS on the positive ion multi-mode. (**a**) Total ion chromatogram of scCO_2_ extract from FDLJ; (**b**) Molecular features of SFE material where different colours indicate different masses.

The extract from the SFE was subjected to untargeted bioactive compounds identification by LC-QToF-MS based metabolomics in positive mode. Mass spectrometry data acquisitions were performed in triplicate (n = 3), see Fig. 4.

Molecular features extraction algorithms were used as a means to extract features from chromatographic data, and 72 features were extracted and generated from positive ionisation mode. Data acquired were aligned and analysis was performed including noise filtering, peak detection, peak deconvolution, retention time alignment, and feature annotation. Molecular formula generator predicted 34 features with putative empirical formula (≥75% MFG score). The list of masses was searched against METLIN Personal Metabolite Database (accessed on May 2018) resulting in 70 and 194 compounds in positive and [M + H-H_2_O] mode with the accuracy tolerance of ≤5 ppm. The experimental masses, retention time, putative empirical formula, error in part per million, number of hits from METLIN database are shown in Table 7. The search indicated that vitamins and phytosterols were likely to be the scCO_2_ extract principles. Further qualitative investigations have been carried out (data not shown) and are beyond the scope of this study.

**Table 7:** Tentative identification of compounds in scCO_2_ extract of leaf juice.

Experimental mass	Retention time (min.)	Putative empirical formula	Error (ppm)@	Number of hits#	Putative compounds$
383.3658	31.77	C_28_H_48_O	5	32	Dihydrobrassicasterol, campesterol
395.3664	31.823	C_29_H_48_O	3	67	Fucosterol, stigmasterol
397.3835	32.698	C_29_H_50_O	0	34	β-Sitosterol
429.3724	27.442	C_29_H_48_O_2_	0	23	Vitamin-d3
431.3885	30.429	C_29_H_50_O_2_	0	11	dl-α-Tocopherol

## General Discussion

In this study, the FDLJ of *C. papaya* was extracted with SFE conditions determined by a 2^6-2^ fractional factorial design. The factors investigated were pressure, temperature, processing time, loading of raw material, sonication and stirring rate. Pressure and temperature were shown to be the main parameters governing supercritical fluid extraction of cytotoxic principles of *C. Papaya* FDLJ. It was found that an increase in pressure results in an increase in the cytotoxicity of the extract, which may indicate that the cytotoxic molecules are lipid-soluble; increasing the pressure leads to a higher density of scCO_2_ and increases the solubility of lipophilic solutes because the distance between the solute and solvent molecules decreases, leading to an increased solubility of lipophilic compounds in scCO_2_^17,18^.

In contrast to the effect of pressure, cytotoxicity was decreased by increasing the temperature from 35 °C to 50 °C. The influence of temperature is more ambiguous than that of pressure as the extraction efficiency of active principles is affected by solvent density and vapour pressure/diffusivity^19^. The decrease in extraction yield (see supplementary dataset 1) and cytotoxicity effect at a higher temperature could be a function of reduced solvent (CO_2_) density and thus a decreased solvation power at a given pressure. In agreement with this hypothesis, another study showed that increasing the temperature to 50 °C led to a lower yield, whereas the temperature of 40 °C was the most appropriate to extract a pyrrolidine alkaloid from the leaf of *Piper amalago*^20^.

One of the advantages of SFC extraction of natural products is a shortened processing time compared to conventional extraction methods. In this study, a processing time of 180 minutes produced an extract with the greatest cytotoxic effect. A longer processing time is likely to prolong the chance of interactions between the solvent and solute thereby enhancing the mass transfer rate.

In order to investigate the amount of material to use for increasing the extraction yield or more ideally, yield of cytotoxic actives, 1 g and 5 g of FDLJ powder were both investigated in the experimental design. The higher amount of starting material had an inappreciable effect on the percentage yield, while increasing the cytotoxic effect (albeit with borderline significance, p = 0.052). This was an interesting result as it suggests that the saturation solubility of the principle components was not reached under the conditions investigated, and that more research may further optimise the SFE process for FDLJ of papaya leaves.

The extraction of FDLJ of *C. papaya* in the present study involved the use of an overhead stirrer and sonicator, both used for agitation of the material by different means. The extraction chamber was equipped with an overhead stirrer in order to improve the rate of mixing and achieve thermodynamic equilibrium. That is, kinetic energy was presumed to increase the rate of miscibility between scCO_2_ solvent and material. The SFE reaction vessel was carefully placed in the same position for each sonication step to help minimise the effects of ‘hot spots’ within the sonicating water bath. The effect of sonication and stirring rate was negligible for yield, however, no stirring did increase the cytotoxicity effect of the extract (P < 0.05). From the interaction plot, there was no observed confounding between sonication and stirring rate or other parameters. The increased cytotoxic effect from stirring, suggests that agitation helped improve the dissolution rate of important molecules.

Most cancer drugs are notoriously cytotoxic to both cancer and normal dividing cells, resulting in side effects for patients. Drugs that selectively target cancer cells while sparing normal cells represent important progress in anticancer therapy. The HaCaT cell line was used together with SCC25 cells to evaluate the cancer cell selectivity of cytotoxicity of the SFE extracts. The SFE extract that showed the most significant selectivity towards cancer cells was FDLJ-SFE-S-E12. Although quantitatively less cytotoxic to the cancer cells than the pre-SFE FDLJ, FDLJ-SFE-S-E12 exhibited a selectivity more marked than that of the FDLJ when comparing extracts obtained from 100 mg of original leaves per mL of medium and showed less toxicity to the non-cancerous cells than that of the FDLJ.

An unexpected finding of our study is that some of the scCO_2_-based SFEs were able to increase the proliferation of non-cancerous HaCaT cells (but not that of the SCC25 cancer cells). This could be explored further in a study trying to optimize the extraction process to increase this feature of the extracts, in search of actives capable of increasing wound healing.

A key advantage of SFE is that it can selectively extract compounds of interest. Supercritical carbon dioxide, as the solvent in SFE, is intrinsically non-polar, which means it is more likely to extract non-polar, lipophilic (hydrocarbon) compounds than more polar compounds. This feature means that the discovery of non-polar cytotoxic compounds from plants is easier than from conventional methods of extraction. Conversely, this very feature may also be problematic if a compound of interest is mostly polar in chemical nature.

In the present study, untargeted UPLC-QTOFMS based metabolomics was employed to assess the composition of scCO_2_ extract of papaya FDLJ. As anticipated, the composition of the SFE was much simpler than that of the FDLJ^15^. Mass spectrometry offers quality analysis by providing accurate mass and putative molecular formula with the potential to narrow down the search against databases. The search revealed the presence, together with other compounds of vitamins and sterols within the scCO_2_ extracts. These were of interest given that stigmasterol, β and γ-sitosterol, and campesterol have been previously identified in the aerial parts of C. papaya^21,22^. Furthermore, some of the tentatively identified molecules have been previously documented to have anticancer properties. Fucosterol, stigmasterol, β and γ-sitosterol, dihydroxybrassicasterol were shown to decrease the viability of cervical cancer HeLa leukaemia (HL-60), colon cancer (SW620), liver cancer (Hep G2), breast cancer BT-474, and tongue squamous carcinoma SCC9 cell line^23–26^. He *et al*. showed that α-tocopherol (vitamin E) suppressed the growth of murine B16 melanomas *in-vitro* and *in-vivo*^27^. It is interesting to note that dl-α-tocopherol stimulated HaCaT wound healing *in-vitro* and exerted protective properties against skin induced tumorigenesis in mice^28,29^.

## Conclusion

This study employed a mathematical factorial design study for scCO_2_ extraction of FDLJ of *C. papaya* and showed pressure, temperature, processing time, loading of material and stirring rate significantly influenced the extraction of cytotoxic actives. In addition, mixed interactions of pressure and temperature, temperature and loading of material significantly influenced the extraction of cytotoxic molecules. The best conditions for extraction of cytotoxic actives were 250 bar, 35 °C, 180 minutes and 5 g of material, and resulted in an extract that was selectively cytotoxic to cancer cells when compared to cells of non-cancer origin. From preliminary qualitative analysis, the potentially active components may be vitamins and phytosterols, and this has been studied - data not shown. Further investigations need to be performed to evaluate the cytotoxicity of individual compounds against SCC25 and HaCaT cells as well as study their mechanism of action.

## Supplementary information

Supplementary Tables
