# Supplementary material for: Factorial design-assisted supercritical carbon-dioxide extraction of cytotoxic active principles from Carica papaya leaf juice
Source: Sci Rep. 2019 Feb 8;9:1716. doi: 10.1038/s41598-018-37171-9 (PMC6368614; doi:10.1038/s41598-018-37171-9)

Supplementary Dataset 1: Yield of SCF extract from FDLJ

Table S1: Experimental matrix for the 2^6-2^ design factors and response for the yield.

| Runs | Factors | | | | | | Response |
| --- | --- | --- | --- | --- | --- | --- | --- |
|  | A | B | C | D | E | F | Yield (%) (R1) |
| 1 | -1 | -1 | -1 | 1 | -1 | 1 | 0.23 |
| 2 | -1 | 1 | 1 | -1 | -1 | -1 | 0.13 |
| 3 | -1 | -1 | -1 | -1 | -1 | -1 | 1.47 |
| 4 | -1 | 1 | 1 | 1 | -1 | 1 | 0.026 |
| 5 | -1 | -1 | 1 | -1 | 1 | 1 | 0.57 |
| 6 | 0 | 0 | 0 | 0 | 0 | 0 | 0.26 |
| 7 | 1 | -1 | -1 | 1 | 1 | 1 | 0.10 |
| 8 | 1 | -1 | -1 | -1 | 1 | -1 | 0.29 |
| 9 | 1 | -1 | 1 | -1 | -1 | 1 | 1.74 |
| 10 | -1 | -1 | 1 | 1 | 1 | -1 | 0.13 |
| 11 | 1 | 1 | -1 | -1 | -1 | -1 | 0.57 |
| 12 | 1 | -1 | 1 | 1 | -1 | 1 | 2.73 |
| 13 | -1 | 1 | -1 | 1 | 1 | 1 | 0.02 |
| 14 | 1 | 1 | 1 | -1 | 1 | 1 | 1.60 |
| 15 | 0 | 0 | 0 | 0 | 0 | 0 | 0.11 |
| 16 | 1 | 1 | -1 | 1 | -1 | -1 | 0.19 |
| 17 | -1 | 1 | -1 | -1 | 1 | 1 | 0.95 |
| 18 | 1 | 1 | 1 | 1 | 1 | 1 | 0.43 |

Table S2: Estimated effects and coefficient for the processing yields (R^2^=99.89%).

| Term | Effect | Coefficient | SE-coefficient | T | P |
| --- | --- | --- | --- | --- | --- |
| Constant |  | 0.7010 | 0.02652 | 26.44 | 0.024 |
| A | 0.5205 | 0.2603 | 0.02652 | 9.81 | 0.065 |
| B | -0.4130 | -0.2065 | 0.02652 | -7.79 | 0.081 |
| C | 0.4470 | 0.2235 | 0.02652 | 8.43 | 0.075 |
| D | 0.4280 | -0.2140 | 0.02652 | -8.07 | 0.078 |
| E | -0.3695 | -0.1848 | 0.02652 | -6.97 | 0.091 |
| F | -0.2380 | -0.1190 | 0.02652 | -4.49 | 0.140 |
| AxB | -0.0945 | -0.0472 | 0.02652 | -1.78 | 0.326 |
| AxC | 0.9005 | 0.4503 | 0.02652 | 16.98 | 0.037* |
| AxD | 0.2505 | 0.1253 | 0.02652 | 4.72 | 0.133 |
| AxE | -0.3230 | -0.1615 | 0.02652 | -6.09 | 0.104 |
| AxF | -0.2445 | -0.1223 | 0.02652 | -4.61 | 0.136 |
| BxC | -0.2080 | -0.1040 | 0.02652 | -3.92 | 0.159 |
| BxF | 0.2570 | 0.1285 | 0.02652 | 4.85 | 0.130 |
| AxBxC | -0.3695 | -0.1848 | 0.02652 | -6.97 | 0.091 |
| AxBxF | -0.1495 | -0.0748 | 0.02652 | 2.82 | 0.217 |
| Centre points |  | -0.5160 | 0.07955 | -6.49 | 0.097 |

*Statistically significant factors (P-value<0.05)

Figure S3: Main effects plot of processing yield. *Statistically significant factors (P-value<0.05).


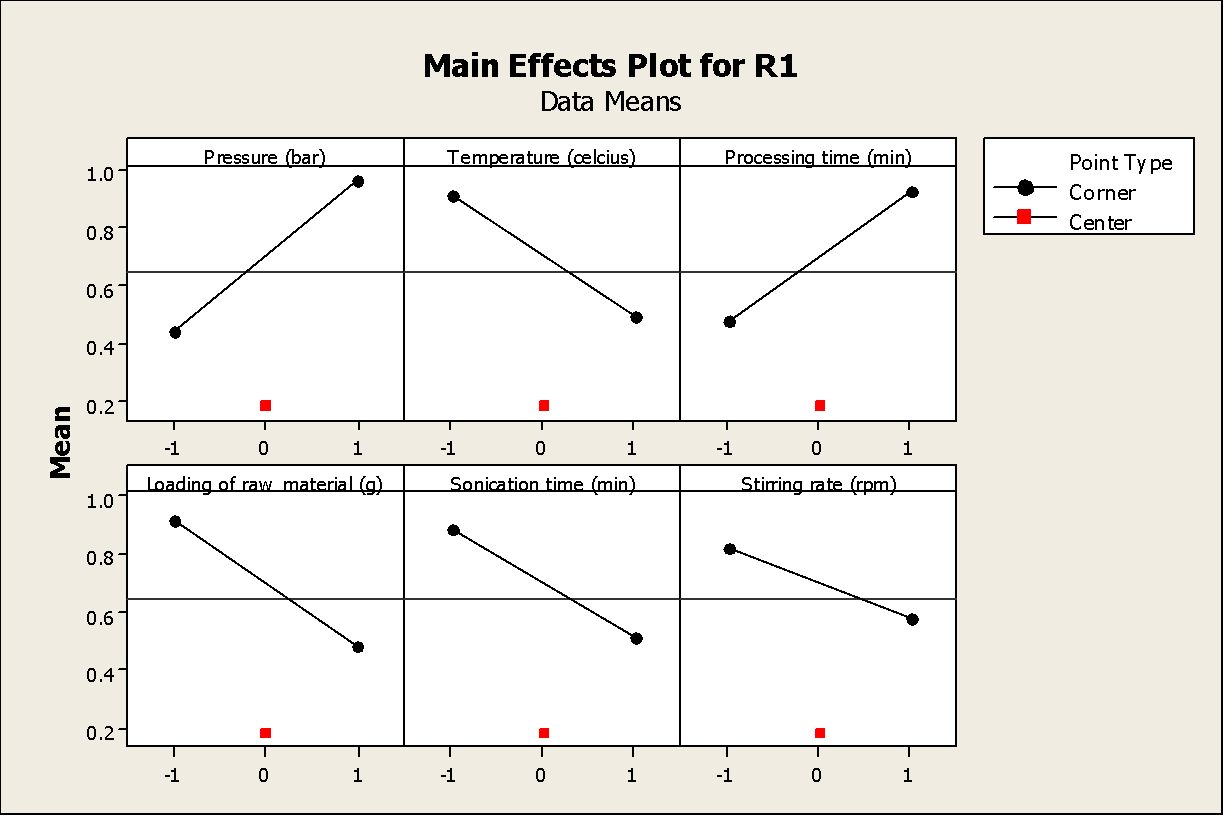

Supplement: Supplementary file 1 — Supplementary Tables [file 41598_2018_37171_MOESM1_ESM.docx]
